# Recognition and management of children and adolescents with conduct disorder: a real-world data study from four western countries

**DOI:** 10.1186/s13034-024-00710-6

**Published:** 2024-01-28

**Authors:** Christian J Bachmann, Oliver Scholle, Mette Bliddal, Susan dosReis, Ingvild Odsbu, Svetlana Skurtveit, Rikke Wesselhoeft, Annika Vivirito, Chengchen Zhang, Stephen Scott

**Affiliations:** 1 Department of Child and Adolescent Psychiatry, University of Ulm, Steinhövelstr. 5, DE-89075 Ulm, Germany; 2 Department of Clinical Epidemiology, Leibniz Institute for Prevention Research and Epidemiology – BIPS, Bremen, Germany; 3 Research Unit OPEN, Department of Clinical Research, University of Southern Denmark, Odense, Denmark; 4 Clinical Pharmacology, Pharmacy and Environmental Medicine, Department of Public Health, University of Southern Denmark, Odense, Denmark; 5 Department of Practice, Sciences, and Health Outcomes Research, University of Maryland School of Pharmacy, Baltimore, MD USA; 6 Department of Chronic Diseases, Norwegian Institute of Public Health, Oslo, Norway; 7 Child and Adolescent Mental Health Odense, Mental Health Services in the Region of Southern Denmark, Odense, Denmark; 8 InGef - Institute for Applied Health Research Berlin GmbH, Berlin, Germany; 9 Department of Child and Adolescent Psychiatry, Institute of Psychiatry, Psychology & Neuroscience, King’s College London, London, UK; 10 National Academy for Parenting Research, King’s College London, London, UK

**Keywords:** Antipsychotics, Comorbidity, Conduct disorder, Hospitalisation, International, Oppositional defiant disorder, Prevalence, Healthcare databases

## Abstract

**Background:**

Conduct disorders (CD) are among the most frequent psychiatric disorders in children and adolescents, with an estimated worldwide prevalence in the community of 2–4%. Evidence-based psychological outpatient treatment leads to significant improvement in about two-thirds of cases. However, there seems to be considerable variation in rates of CD diagnoses and implementation of evidence-based interventions between nations. The aim of this study was to compare administrative prevalence and treatment patterns for CD in children and adolescents seen in health care systems across four Western countries (Denmark, Germany, Norway, and the USA).

**Methods:**

Cross-sectional observational study using healthcare data to identify children and adolescents (aged 0–19 years) with an ICD-10 code for CD within the calendar year 2018. Within each country’s study population, the prevalence of CD, psychiatric comorbidity, psychopharmacological treatment, and psychiatric hospitalisation was calculated.

**Results:**

The prevalence of diagnosed CD differed 31-fold between countries: 0.1% (Denmark), 0.3% (Norway), 1.1% (USA) and 3.1% (Germany), with a male/female ratio of 2.0–2.5:1. The rate of psychiatric comorbidity ranged from 69.7 to 86.1%, with attention-deficit/hyperactivity disorder being most common. Between 4.0% (Germany) and 12.2% (USA) of youths with a CD diagnosis were prescribed antipsychotic medication, and 1.2% (Norway) to 12.5% (Germany) underwent psychiatric hospitalisation.

**Conclusion:**

Recognition and characteristics of youths diagnosed with CD varied greatly by country. In some countries, the administrative prevalence of diagnosed CD was markedly lower than the average estimated worldwide prevalence. This variation might reflect country-specific differences in CD prevalence, referral thresholds for mental health care, diagnostic tradition, and international variation in service organisation, CD recognition, and availability of treatment offers for youths with CD. The rather high rates of antipsychotic prescription and hospitalisation in some countries are remarkable, due to the lack of evidence for these therapeutic approaches. These findings stress the need of prioritising evidence-based treatment options in CD. Future research should focus on possible reasons for inter-country variation in recognition and management of CD, and also address possible differences in patient-level outcomes.

## Background

In ICD-10/-11, conduct disorder (CD) is a term encompassing both Oppositional Defiant Disorder (ODD) which is usually milder and seen in younger children, and Conduct Disorder, which involves more severe violations of societal norms and is more common in later childhood and adolescence. Typical oppositional defiant behaviours include loses temper, easily annoyed, angry and resentful, refuses to comply with requests, blames others for mistakes, etc. Typical CD symptoms include starting fights, carrying a weapon, physically cruel, forcing someone into sexual activity, setting fires, etc. [1]. Despite having very similar antecedents and life courses, they are considered separate disorders in DSM-IV/-5. In this paper, the term CD will be used to refer to both variants.

CD is amongst the most common of child and adolescent psychiatric disorders [2, 3], with an estimated worldwide prevalence in the community of 2–4% [4–6]. There is a high rate of comorbid psychiatric conditions, particularly attention-deficit/hyperactivity disorder (ADHD) [7]. Affected children and their families experience considerable distress, often accompanied by social and educational impairment (e.g., school dropout, social exclusion). Without treatment, early onset CD in particular tends to also affect adolescent life, where about 50% go on to engage in drug misuse and conduct criminal offenses [8, 9]. Childhood CD further predicts homelessness, poor physical and mental health, and excess mortality in adulthood [10–13]. The potential of negative clinical and psychosocial long-term outcomes highlights the impact of CD, and the importance of early detection and intervention. CD also has economic implications: Child-level research has shown that by age 28, a group of children with CD had costs nine times higher than those without any behaviour problems (70,019 GBP vs. 7,423 GBP), with the majority of costs being attributed to criminal activity [14]. Two European studies showed that children with a CD diagnosis or a high level of conduct problems had four-fold higher service use costs than children without a CD diagnosis, or a low level of conduct problems [15, 16].

Most guidelines [17–20] recommend psychosocial interventions or parent training as the first line of treatment in younger children with CD [21, 22]. In older children or in adolescents, multi-component and multimodal treatment approaches are indicated and cost-effective [23–25]. The vast majority of CD cases can be managed in an outpatient setting [18], and there is no evidence for the short- or long-term effectiveness of inpatient treatment [1]. No psychopharmacological agent is currently licensed in any country for the treatment of CD. Antipsychotic treatment in CD is recommended only in youths with very high levels of impulsivity, when other treatment options have been exhausted [18]. Notably, in recent years the utilisation of antipsychotics for children and adolescents with CD has been increasing, with antipsychotic treatment rates of up to 20% in some Western countries [26–31]. This constitutes an issue of serious concern because of the metabolic adverse events (e.g. weight gain, type 2 diabetes mellitus [32, 33]) and the limited evidence base for use in CD [34].

Cross-national comparisons of youths with CD using real-world data may provide important information on international variation in the recognition and management of CD, and have the potential to identify the utilisation of evidence-based vs. non evidence-based treatment options. Therefore, this study aimed to compare prevalence and treatment patterns of paediatric CD across four Western countries (i.e., Denmark, Germany, Norway, and the USA) based on administrative data.

## Methods

We used 2018 data for children and adolescents aged 0–19 years from Denmark, Germany, Norway, and the USA to conduct a cross-sectional observational study. The age range was chosen to ensure comparability with other international studies (e.g. [35]). All diagnoses were coded according to the 10th revision of the International Statistical Classification of Diseases and Related Health Problems (ICD-10), and prescribed drugs were coded according to the Anatomical Therapeutic Chemical (ATC) classification [36]. A common data protocol guided the analyses in all countries.

### Data sources

#### Denmark

Danish data were derived from the Danish National Patient Registry, which holds information on hospital contacts in Denmark for all Danish citizens (about 5.8 million in 2018) [37]. Using the unique identification number assigned to all individuals in Denmark at birth or first immigration, we linked data to the Danish National Prescription Registry [38] on filled prescriptions. The National Prescription Registry holds no information on indication for prescription. Underlying annual population counts by sex and age were obtained from national census data (statistikbanken.dk). Diagnoses assigned by a general practitioner, or a private practicing psychiatrist are not available in the Danish data. However, the proportion of young individuals using private specialist physicians in Denmark is limited due to the free of charge access to public specialist health care; with a recent study demonstrating that more than 86% of ADHD cases were diagnosed within the public health system [39].

#### Germany

The German data comprised administrative claims from the InGef (Institute for Applied Health Research Berlin GmbH) research database. The database covers longitudinal data of approximately nine million Germans insured from 2013 to 2021 in one of approximately 60 statutory health insurances. In addition to sociodemographic information, the database contains information about outpatient services and diagnoses, hospital data including admission periods, main and secondary diagnoses and procedures conducted, prescription drug data, information on prescribed aids and remedies, and the costs accrued in these sectors. For this study, a sample containing approximately 4.8 million persons representative to the age and sex structure of the total German population was used. The sample database shows good overall agreement with the German population on measures of morbidity, mortality, and drug usage [56].

#### Norway

Data from the Norwegian Patient Registry and the Norwegian Prescription Database were linked via the unique personal identification number. The Norwegian Patient Registry contains information from all citizens in Norway (about 5.3 million in 2018) on all patient contacts with public specialist health care services, including private institutions and medical specialists contracted to the regional health authorities [57]. Diagnoses assigned by primary health care providers were not available in the Norwegian data. The Norwegian Prescription Database contains information on all dispensed prescription drugs from all pharmacies in Norway [58]. The denominator for the prevalence analysis was extracted from Statistics Norway.

#### United States of America

The study population was selected from IQVIA PharMetrics® Plus for Academics health plan claims data for 2018. The PharMetrics Plus for Academics data contain information on medical encounters in inpatient and outpatient settings and on prescription drugs dispensed in outpatient pharmacies. The data are for 10 million individuals that are representative to the age and sex composition of commercially insured individuals in the USA. In the USA, about two thirds of children aged 0–17 years have a commercial health insurance, with the commercially insured population having lower rates of chronic health conditions than their publicly insured peers [59].

Inpatient and outpatient claims record the date of service, place of service, codes for procedures performed, and diagnosis codes. Pharmacy claims include the National Drug Codes (NDC), which identifies the medication name and unit strength, the quantity dispensed, and the days supply. The NDC is a universal coding system that assigns a unique identifier for all licensed drugs marketed in the USA. To align with the data from European countries, the NDCs were converted to 5th level ATC codes based on therapeutic class and generic name. Unique hospitalisations were estimated from the inpatient claims for all services provided during the hospitalisation. Service dates that were four or more days apart were counted as separate hospitalisations. The service date on the last claim in an encounter was the discharge date, which was used to estimate the duration of the hospitalisation.

### Study population and measures of interest

The eligible population was all individuals aged 0–19 years in the calendar year 2018; with continuous observation time from January 1 to December 31, 2018; valid information on sex; and residence inside the country (i.e., denominator).

From this population, we selected all individuals who had at least one documented inpatient or outpatient CD diagnosis. This included ICD-10 codes F91 (“Conduct disorders”; which includes F91.0 (“CD confined to the family context”), F91.1 (“Unsocialized CD”), F91.2 (“Socialised CD”), and F91.3 (“Oppositional defiant disorder” (ODD); a diagnosis usually made in children up to age 10 years)), F90.1 (“Hyperkinetic conduct disorder”; a combined diagnosis of CD plus attention deficit/hyperkinetic disorder), or F92 (“Mixed disorders of conduct and emotions”; a combined diagnosis of CD and an emotional disorder) in the calendar year 2018. This comprised the study target population.

The prevalence of CD was defined as the proportion of individuals with CD per 100 individuals in the eligible population (referred to as denominator above). The number of hospitalisations with a diagnosis of CD was defined as the number of unique inpatient admissions in 2018 with any diagnosis of CD—determined by the number of different days of admission to hospital between January 1 and December 31, 2018. The sum of days of hospitalisations with a diagnosis of CD was defined as the sum of days from admission to discharge (or December 31, 2018, whichever occurred first) from inpatient stays with any diagnosis of CD in 2018.

### Data analysis

For each country’s study population, we determined the population prevalence of CD in 2018, overall and stratified the CD cases by sex (male, female), and by age groups (0–4, 5–9, 10–14, 15–19 years). Among children and adolescents with a CD diagnosis, we assessed comorbid psychiatric disorder diagnoses overall and by diagnosis groups according to Dalsgaard et al. [61]. Additionally, we determined the proportion of children and adolescents with a CD diagnosis who had at least one dispensed prescription for a psychopharmacologic drug, overall and by therapeutic class, i.e., antipsychotics (ATC code: N05A), phenothiazine derivates (R06AD), antidepressants (N06A), psychostimulants (N06BA), anxiolytics/hypnotics/sedatives (N05B/N05C, without N05CH01 (melatonin); referred to as “tranquillisers”), opioids (N02A), antiepileptics/ mood stabilisers (N03A). Finally, among those with a hospitalisation related to CD, we calculated the average number of days per hospitalisation by dividing the sum of days of hospitalisations by the unique number of hospitalisations due to CD.

## Results

Table 1 shows the population prevalence of CD, which ranged from 0.1% (Denmark) to 3.1% (Germany). The male/female ratio was 2.0–2.5:1.

**Table 1:** Population prevalence of conduct disorder diagnoses in 0–19 year olds, 2018 (in %)

		Denmark (*N* = 1,306,550)	Germany (*N* = 732,020)	Norway (*N* = 1,225,424)	USA (*N* = 123,971)
Overall		0.11	3.05	0.23	1.10
Sex	Male	0.15	4.05	0.33	1.47
	Female	0.06	1.98	0.13	0.72
Age group (in years)	0–4	0.01	1.73	0.01	0.27
	5–9	0.10	4.12	0.23	1.43
	10–14	0.17	4.27	0.40	1.55
	15–19	0.14	2.15	0.25	0.92

Conduct disorder ICD-10 subtypes by country are shown in Table 2, with ODD being most common in the USA, and hyperkinetic CD most frequent in Denmark and Norway.

**Table 2:** Characteristics of children and adolescents with a conduct disorder in 2018 (in %)

		Denmark (*N* = 1,385)	Germany (*N* = 22,324)	Norway (*N* = 2,879)	USA (*N* = 1,367)
Sex	Male	70.4	68.4	72.6	68.1
	Female	29.6	31.6	27.4	31.9
Age group (in years)	0–4	1.5	14.2	1.0	4.3
	5–9	23.1	32.8	25.9	32.2
	10–14	41.3	34.7	44.7	39.0
	15–19	34.1	18.3	28.3	24.5
Diagnostic subtype*	Oppositional defiant disorder (ODD) (F91.3)	13.9	16.3	18.8	37.0
	Conduct disorder, excluding ODD (F91, excluding F91.3)	15.8	46.0	18.2	28.5
	Hyperkinetic conduct disorder (F90.1)	37.6	20.2	35.8	34.5
	Mixed disorders of conduct and emotions (F92)	34.2	17.4	27.2	N/A**

*Mutually exclusive according to the hierarchy from top to bottom

** Not applicable, as this code is not included in ICD-10-CM

**Fig. 1:**
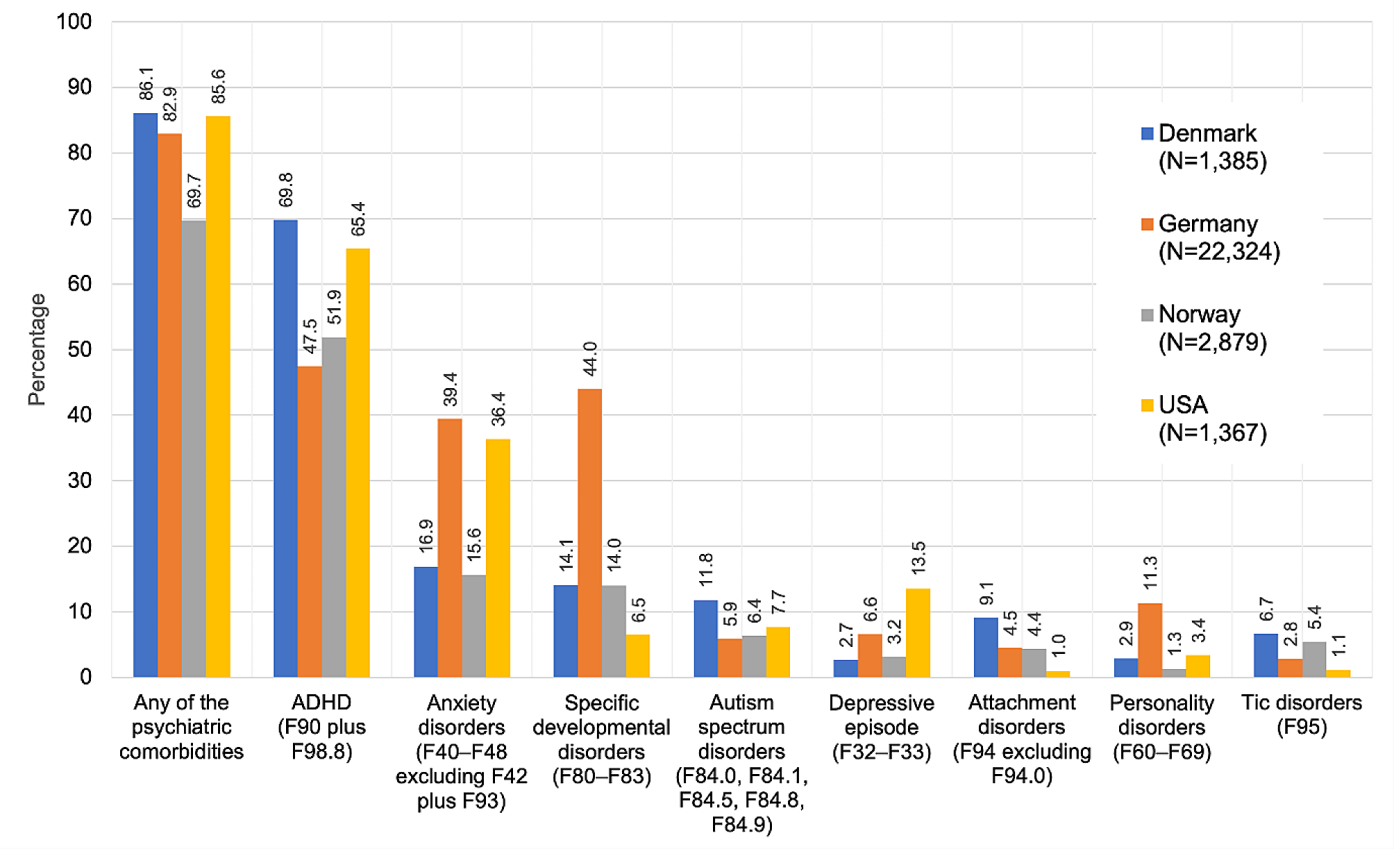
Most frequent psychiatric comorbidities among children and adolescents with a conduct disorder diagnosis in 2018 (in %)

The percentage of psychiatric comorbidity in children and adolescents with CD ranged from 70 to 86%, with ADHD being the most common in all countries (Fig. 1).

Comorbidity profiles differed by sex, with females showing higher rates of depression, anxiety disorders, eating disorders, and personality disorders, and males having higher rates of ADHD, specific developmental disorders, and tic disorders (Table 3).

**Table 3:** Psychiatric comorbidities among children and adolescents with a diagnosis of conduct disorder in 2018 (in %)

	Total				Sex							
					Male				Female			
	DK	GER	NOR	USA	DK	GER	NOR	USA	DK	GER	NOR	USA
Total N	1,385	22,324	2,879	1,367	975	15,274	2,089	931	410	7,050	790	436
Any of the psychiatric comorbidities	86.1	82.9	69.7	85.6	88.1	85.5	70.1	86.3	81.5	77.2	68.6	84.2
Substance use disorders (F10–F19)	7.5	2.5	2.7	2.4	7.6	2.0	2.3	2.7	7.3	3.4	3.8	1.8
Schizophrenia spectrum disorder (F20–F29)	2.7	0.5	0.4	1.8	1.8	0.4	0.2	1.7	4.6	0.6	0.8	2.1
Mood disorders (F30–F39)	3.0	7.9	4.3	20.4	1.7	6.0	2.9	18.9	5.9	11.9	8.1	23.6
Bipolar disorder (F30–F31)	*n* < 5	0.1	0.7	3.2	*n* < 5	0.1	0.5	2.4	*n* < 5	0.1	1.3	4.8
Depressive disorder (F32–F33)	2.7	6.6	3.2	13.5	1.5	4.8	2.0	11.5	5.6	10.5	6.3	17.9
Anxiety disorders (F93, F40–F48, excluding F42)	16.9	39.4	15.6	36.4	12.3	37.0	14.0	33.7	25.6	44.8	19.7	42.0
Obsessive-compulsive disorder (F42)	2.2	1.1	1.2	1.4	1.8	1.1	1.1	1.1	2.9	1.2	1.5	2.1
Eating disorders (F50)	0.6	2.0	0.8	0.7	N/A (*n* < 5)	1.2	0.3	0.5	1.7	3.7	2.0	0.9
Personality disorders (F60–F69)	2.9	11.3	1.3	3.4	1.1	10.7	0.5	3.1	7.1	12.6	3.3	3.9
Mental retardation (F70–F79)	5.7	4.8	2.4	1.0	6.7	5.2	2.3	0.9	3.4	3.9	2.5	1.4
Specific developmental disorders (F80–F83)	14.1	44.0	14.0	6.5	15.6	47.8	15.0	6.9	10.5	35.7	11.4	5.7
Autism spectrum disorders (F84.0, F84.1, F84.5, F84.8, F84.9)	11.8	5.9	6.4	7.7	11.8	7.3	7.0	9.6	12.0	2.8	4.7	3.7
ADHD (F90, F98.8)	69.8	47.5	51.9	65.4	76.1	54.4	54.1	70.0	54.9	32.6	46.1	55.7
Attachment disorders (F94, excluding F94.0)	9.1	4.5	4.4	1.0	9.2	4.6	4.7	1.0	8.8	4.3	3.7	0.9
Tic disorders (F95)	6.7	2.8	5.4	1.1	8.2	3.4	6.4	1.4	3.2	1.7	5.4	0.5

**Fig. 2:**
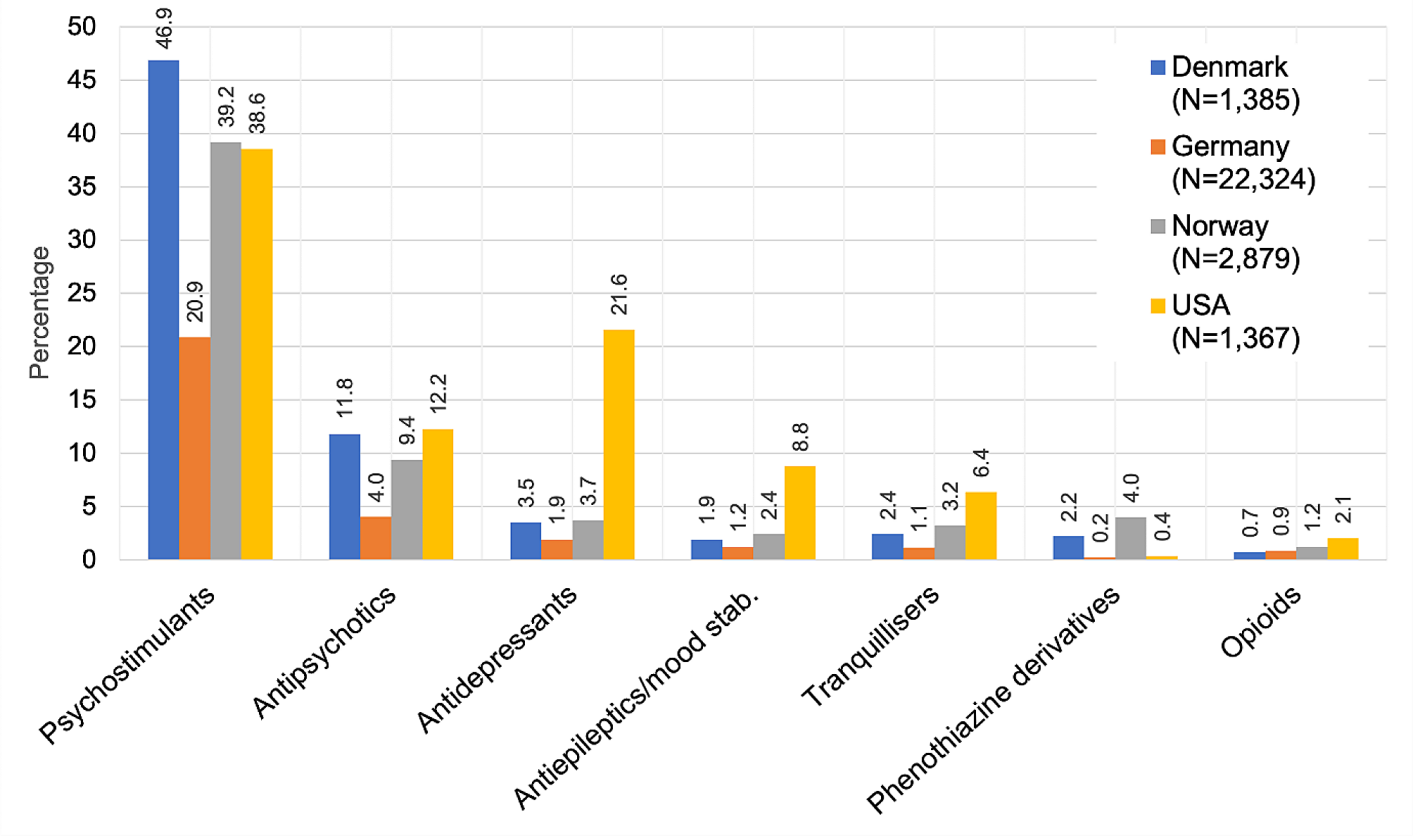
Psychopharmacological treatment among children and adolescents with a conduct disorder diagnosis in 2018 (in %)

Regarding psychopharmacotherapy, psychostimulants were prescribed most frequently, followed by antipsychotics, antidepressants, and antiepileptics/mood stabilisers (Fig. 2).

The proportion of children and adolescents diagnosed with CD who were prescribed antipsychotic medication ranged from 4.0 to 12.2%. Prescribing of psychostimulants and antipsychotics to children and adolescents diagnosed with CD in Denmark, Norway, and USA was substantially higher compared to Germany. In all countries, risperidone was more frequently prescribed than aripiprazole (risperidone and aripiprazole prevalence, respectively, was: Germany: 2.4% vs. 0.5%, Denmark: 3.5% vs. 2.7%, Norway: 5.3% vs. 1.7%, US: 5.6% vs. 4.6%). Regarding psychiatric hospitalisation, rates ranged from 1.2 to 12.5%, with a mean duration per stay of 2.7 days to 22.3 days (Table 4).

**Table 4:** Treatment utilisation in children and adolescents with a CD diagnosis in 2018

	Denmark (*N* = 1,385)	Germany (*N* = 22,324)	Norway (*N* = 2,879)	USA (*N* = 1,367)
Percentage of CD patients with at least one hospitalisation	8.9%	12.5%	1.2%	6.9%
Number of hospitalisations with a diagnosis (primary/secondary) of CD per 1,000 persons	114.8	241.0	11.5	89.0
Hospitalisation days with a diagnosis of CD (primary/secondary) per 1,000 persons	1627.4	5378.0	226.8	243.0
Average number of days per hospitalisation	14.2	22.3	19.7	2.7

## Discussion

### Prevalence of CD

The population prevalence of youths diagnosed with CD in this study differed 30-fold between the Scandinavian countries Denmark and Norway with the lowest prevalence and Germany which had the highest prevalence.

This variation may partly be explained by actual differences in CD prevalence between countries [1]: Scandinavian countries have previously shown lower prevalence rates of various psychiatric disorders compared to other Western countries [40]. For example, the prevalence of psychiatric disorders based on structured diagnostic interviews among preschoolers in Norway was lower than in the USA [41]. Similarly, Norwegian 10–14-year-olds had a lower prevalence of psychiatric disorders (based on a web-based psychiatric interview; DAWBA) than published worldwide prevalence estimates [42]. In contrast to these findings, a Danish study from 2006 revealed a prevalence of 5% for CD/ODD according to DSM-IV (assessed with the K-SADS-PL) among 8- to 9-year-old children [43].

The CD prevalence in the Scandinavian countries included in our study was far below the CD prevalence estimate of 2–4% in a meta-analysis by Polanczyk et al. of 28 studies worldwide [4]. While this meta-analysis, however, only included one Scandinavian study, where diagnoses were assigned based on an online survey [44], Polanczyk et al. concluded that the majority of the heterogeneity in their prevalence estimates could be explained by differences in methodology rather than by geographic location of studies. Nevertheless, the Norwegian studies mentioned above reported prevalence rates of mental disorders that were relatively lower than other countries, leading the authors to coin the concept of “the Nordic advantage in child mental health” [45].

Another possible explanation is that youths with CD are not seen in secondary mental health services due to the organisation of the health system. For example, Denmark and Norway hold a long tradition for primary care service use, meaning that not all children with CD might attend child and adolescent mental health services (CAHMS) and be assigned with an official diagnosis. This might have led to an underestimation of the Danish and Norwegian prevalence proportions registered in our study. Nevertheless, recent data shows that for Danish children with ADHD (which has a considerable overlap with CD), about 86% were diagnosed in mental health services [39], suggesting that the role of primary care in diagnosing CD would also be negligible. The relatively good provision of CAMHS in both mentioned countries also implies that a lack of mental health professionals’ capacity does probably not constitute a significant barrier towards the identification of CD cases. Germany, on the other hand, has a more extensive use of inpatient treatment compared to the other countries, which probably contributes to the high CD prevalence proportion there.

Also, there are no available clinical treatment guidelines for CD among youths in Denmark and Norway. This may contribute to lower awareness of CD and/or higher thresholds for referral to mental health care, which in turn would lead to under-recognition and subsequently to lower diagnostic rates. Moreover, even when using consistent guidelines, the prevalence between CAMHS may differ up to 5-fold [46].

Apart from the inter-country differences in overall CD population prevalence, our findings showed a male predominance and that the age group of 10–14 year olds were more commonly diagnosed with CD across countries, which is in accordance with the literature [1].

### Treatment of children with CD

Treatment utilisation in children and adolescent with a diagnosis of CD differed significantly between countries, especially regarding prescription of antipsychotics, and hospitalisation.

The medication group most frequently prescribed were psychostimulants, which is in line with the high proportion of children and adolescents with comorbid ADHD. The second most common group of prescribed psychotropic medication were antipsychotics. The higher use of antipsychotics and stimulants in youths diagnosed with CD in the Scandinavian countries could be due to these countries only treating the more severe CD cases in a hospital setting. Antipsychotic prescription was lowest among youths with CD in Germany. One possible explanation is that Germany holds a long tradition for inpatient mental health care [47], which is underlined by Germany having the highest number of psychiatric inpatient beds per 100,000 children and adolescents (Table 5; [48]). Despite the lack of evidence for inpatient treatment in CD [1], this is a common treatment choice in Germany, where youths with CD constitute nearly 20% of all child and adolescent psychiatric inpatients [3].

This proposed explanation is supported by our findings, where German youths with CD had the longest hospital stays of all four countries. In contrast, the lower hospitalisations rates in Denmark and Norway might be explained through the better availability of evidence-based parenting programs for children and adolescents with CD in these countries [49, 50]. The short duration of inpatient treatment in the USA is in line with the generally shorter length of hospitalisations in the USA health system [51].

While parent training (e.g. The Incredible Years, Triple P) constitutes an important, evidence-based treatment option for children with CD up to 12 years of age, our data set did not include information on the utilisation of parent training, as these trainings are often provided and/or reimbursed by social services, and not by health insurance funds. This fact, together with the circumstance that parent trainings are much better available in Scandinavian countries, might also have led to an underestimation of CD prevalence rates in Denmark and Norway, as some families might just have attended parent trainings without any further contact with (mental) health services.

**Table 5:** National frameworks for the management of conduct disorder

	Denmark	Germany	Norway	USA
Clinical guideline for the management of CD available	No	Yes [17]	No	No
Guideline quality	N/A	High	N/A	N/A
Psychiatric inpatient beds per 100,000 youths [48, 52]	18.5	64.0	27.5	29.7 (incl beds for adults)
Child psychiatrists per 100,000 youths [48, 53, 54]	10.3	8.0	38.0	9.8
Indication for psychopharmacotherapy	N/A	Severe cases with high impulsivity	N/A	N/A
Indication for psychiatric inpatient treatment	N/A	Suicidality, major comorbidity	N/A	N/A

### Psychiatric comorbidity in children with CD

In our study, children and adolescents with CD showed high rates of psychiatric comorbidity, with ADHD being the most common comorbidity in all countries. This finding is in line with the existing literature, especially the recent study by Konrad et al. [7], which is based on a multi-centre clinical sample of children and adolescents with CD. In their study, 85.8% of boys and 87.9% of girls had any current psychiatric comorbidity (with ADHD being the most common comorbidity), and 89.2% of boys/ 93.4% of girls had any lifetime comorbidity. These numbers correspond well with the one-year-prevalence of 69.7–86.1% found in our study. In terms of sex differences in comorbidity profiles, we found a higher prevalence of internalising disorders and personality disorders in females, and a higher prevalence of ADHD, specific developmental disorders, and tic disorders in males. This pattern correlates well with sex-specific distribution rates of psychiatric disorders in the general population, and also with the findings of Konrad et al. [7], who also found higher rates of depression, anxiety disorders, and (borderline) personality disorders in females with CD, and higher rates of ADHD and tics in males with CD. In all countries in our study, males had slightly higher comorbidity rates.

While the overall psychiatric comorbidity rates in the studied countries were relatively similar, there were some notable country-specific differences, especially with regard to diagnostic rates of ADHD, and anxiety disorders. Regarding the high prevalence of ADHD in the USA, this is in line with the high prevalence known from other studies [55], while the difference in anxiety disorder diagnoses cannot be sufficiently explained.

### Strengths and limitations

This study is the first to compare population prevalence and treatment patterns of paediatric CD between countries based on real-world data. As the study employed nationally representative data bases, the results are generalisable to the publicly insured population of Denmark, Germany, and Norway, and for the commercially insured US population [37, 38, 56–58]. Also, by using secondary data, any recall bias was avoided.

Limitations of the study include the lack of information regarding the validity of the coded diagnoses (e.g. whether the diagnoses were coded according to clinical examination, or standardised diagnostic instruments). Also, the secondary data employed in this study did not include information on the indications for prescribed drugs, thus precluding definitive conclusions regarding the appropriateness of antipsychotic prescriptions in children with a CD diagnosis. Moreover, as commercially insured US youths have less chronic health conditions than privately insured youth (15.3% vs. 22.9%) [59], the figures presented in this paper probably underestimate CD rates for the whole US youth population. Another limitation is the lack of data on parent training as an evidence-based treatment option. Finally, data on socio-economic status, ethnicity and region of residence (rural vs. urban) were not available for all four studied countries, which constitutes a limitation.

## Conclusions

In this study, the prevalence proportions of 0–19-year-olds diagnosed with CD varied greatly between Germany, Norway, Denmark, and the US. In the two Scandinavian countries, the population prevalence of diagnosed CD was markedly lower than prevalence estimates reported in a world-wide meta-analysis. These findings might reflect country-specific differences in the existence of CD among youths, which might be lower in the Scandinavian countries. They are, however, also likely to reflect national variation in recognition and management of these patients. Our findings highlight the heterogeneity of recognition and management of CD in Western countries, which might be associated with variation in available treatment options, especially parent training. Also, in some countries treatment options are common which are not in line with current evidence. This finding stresses the need of prioritising evidence-based treatment options in CD.

Future research should focus on possible reasons for inter-country variation in recognition and management of CD (including the role of clinical guidelines [60]), and also address possible differences in patient-level outcomes.

## Data Availability

Not applicable. As the authors are not the owners of the data, they are not legally entitled to grant access to the research databases used for this study. The IQVIA PharMetrics® Plus for Academics closed claims data obtained under license from IQVIA Inc. The raw data cannot be publicly shared since it was obtained from IQVIA and as per signed agreement between University of Maryland Baltimore and IQVIA Inc. All relevant data in the manuscript that supports the research objectives and conclusions are provided. For further information on data access, please contact IQVIA.

## Abbreviations

ADHD: Attention-deficit/hyperactivity disorder
ATC: Anatomical Therapeutic Chemical classification
CAMHS: Child and adolescent mental health services
CD: Conduct disorder
DSM-IV/-5: Diagnostic and Statistical Manual of Mental Disorders, 4th/5th edition
ICD-10/-11/-CM: International Statistical Classification of Diseases and Related Health Problems, 10th Revision/ 11th Revision/ Clinical Modification
NDC: National Drug Codes
ODD: Oppositional-defiant disorder
OTC: Over-the-counter
